# Various Uses for Ammonia

**Published:** 1890-02

**Authors:** Ray Joyce


### VARIOUS USES FOR AMMONIA.

A little ammonia in tepid water will soften and cleanse the skin.

Spirits of ammonia inhaled, will often relieve a severe headache.

Doorplates should be cleaned by rubbing with a cloth wet in ammonia and water.

If the color has been taken out of silks by fruit stains, ammonia will usually restore the color.

To brighten carpets wipe them with warm water in which has been poured a few drops of ammonia.

One or two tablespoonfuls of ammonia added to a pail of water will clean windows better than soap.

A few drops in a cupful of warm water, applied carefully will remove spots from paintings and chromos.

When acid of any kind gets on clothing, spirits of ammonia will kill it. Apply chloroform to restore the color.

Keep nickel, silver ornaments and mounts bright by rubbing with a woolen cloth saturated with spirits of ammonia.

Grease spots may be taken out with weak ammonia in water; lay soft white paper over and iron with a hot iron.

Ammonia applied two or three times on a fresh cold-sore will kill it. It will drive it away if used when the cold-sore is first felt.

A tablespoonful of ammonia in a gallon of warm water will often restore colors in carpets ; it will also remove white-wash from them.

Yellow stains, left by sewing machine oil, on white, may be removed by rubbing the spot with a cloth wet with ammonia, before washing with soap.

Equal parts of ammonia and turpentine will take paint out of clothing, even if it be hard and dry. Saturate the spot as often as necessary, and wash out in soap suds.

If those who perspire freely, would use a little ammonia in the water they bathe in every day, it would keep their flesh sweet and clean, doing away with any disagreeable odor.

Old brass may be cleaned to look like new by pouring strong ammonia on it, and scrubbing with a scrub-brush; rinse in clear water.

Put a teaspoonful of ammonia in a quart of water, wash your brushes and combs in this, and all grease and dirt will disappear. Rinse, shake and dry in the sun, or by the fire.

Flannels and blankets may be soaked in a pail of water containing one tablespoonful of ammonia and a little suds. Rub as little as possible and they will be white and clean, and will not shrink.

One teaspoonful of ammonia to a teacupful of water will clean gold or silver jewelry ; a few drops of clear aqua ammonia poured on the underside of diamonds, will clean them immediately, making them very brilliant.—RAY JOYCE in *Good Housekeepimg.*

